# Supplementary figures and images for: Phylogenomics from transcriptomic “bycatch” clarify the origins and diversity of avian trypanosomes in North America
Source: PLoS One. 2020 Oct 8;15(10):e0240062. doi: 10.1371/journal.pone.0240062 (PMC7544035; doi:10.1371/journal.pone.0240062)

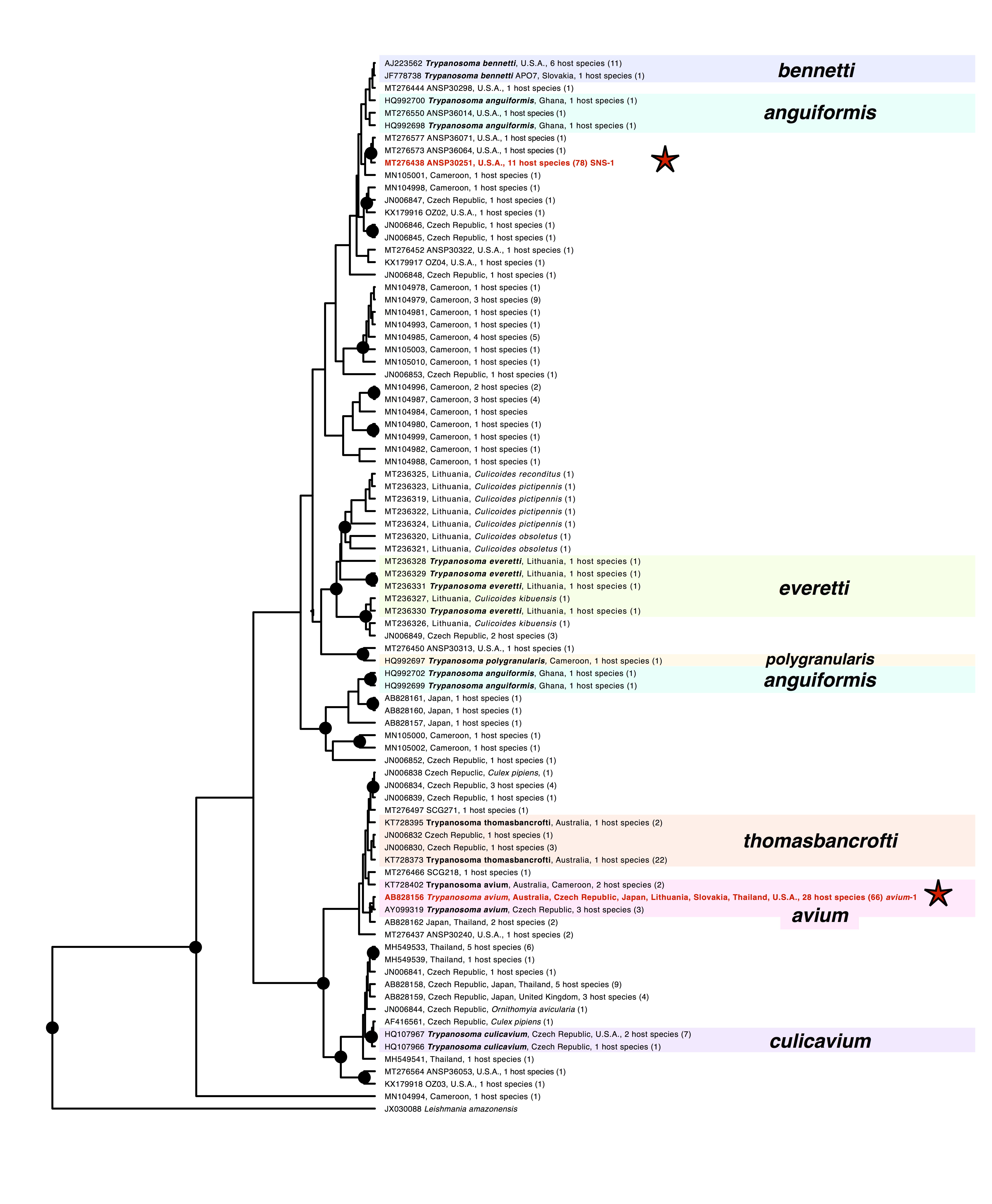

Supplement: S1 Fig — Haplotypes avium-1 and SNS-1 are highlighted in red and marked with a star. (TIFF) [file pone.0240062.s001.tiff]

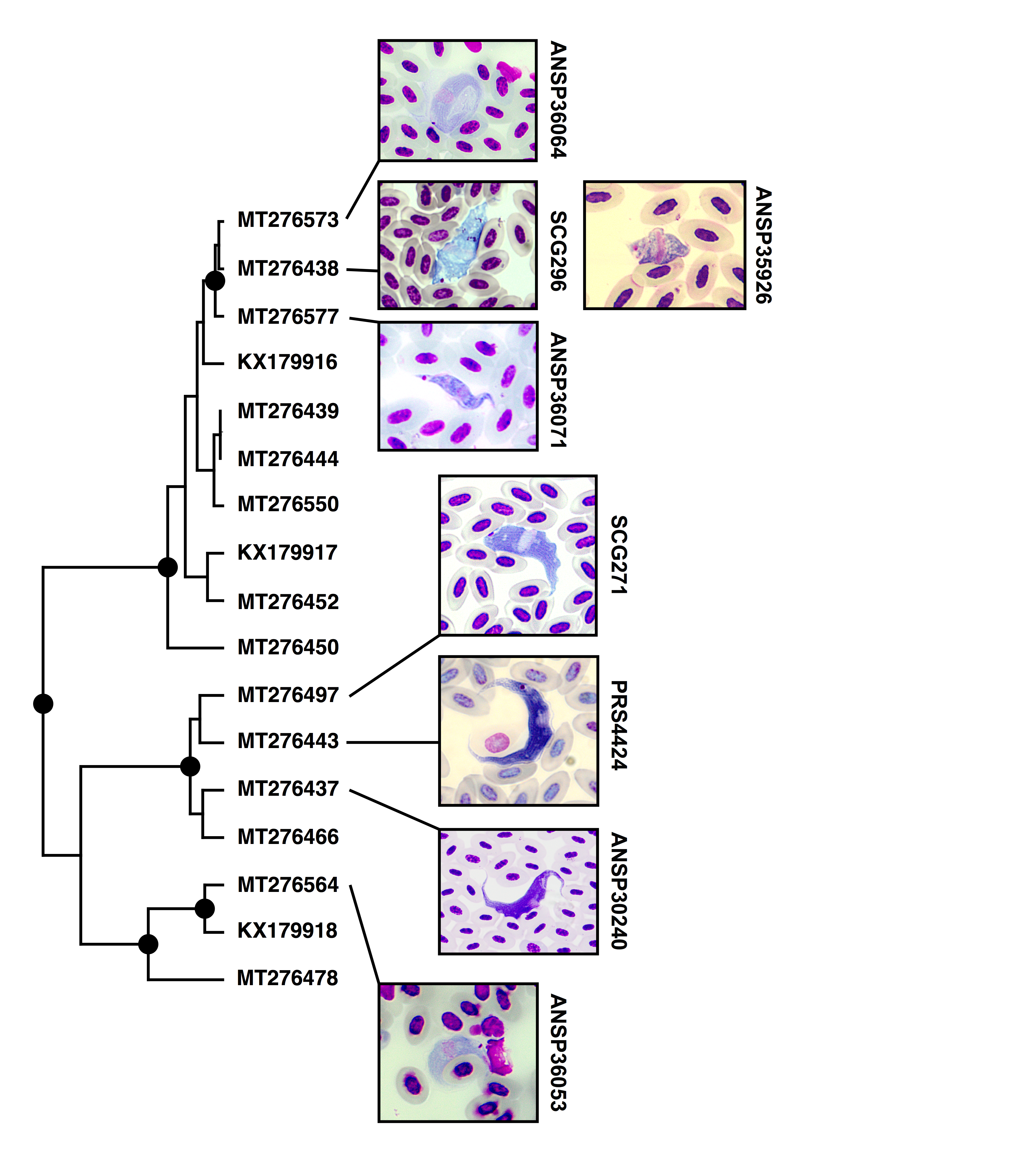

Supplement: S2 Fig — Shown are microscopic images that correspond to the trypanosome 18S haplotypes in the gene tree to the left. Isolate sample numbers are shown to the right of each image. (TIFF) [file pone.0240062.s002.tiff]
