# Supplementary material for: Phylogenomics from transcriptomic “bycatch” clarify the origins and diversity of avian trypanosomes in North America
Source: PLoS One. 2020 Oct 8;15(10):e0240062. doi: 10.1371/journal.pone.0240062 (PMC7544035; doi:10.1371/journal.pone.0240062)
Supplement: S1 Table — Documented host species for Trypanosoma 18S haplotypes avium-1 and SNS-1 from this study and previously sequenced isolates on GenBank. (DOCX) [file pone.0240062.s003.docx]

**S1 Table.** Documented host species for *Trypanosoma* 18S haplotypes *avium*-1 and SNS-1 from this study and previously sequenced isolates on GenBank. Hosts that are insect vectors are listed in bold.

| **Haplotype** | **Isolate and host species** |
| --- | --- |
| *avium*-1 | AB828156 Coma1 *Corvus macrorhynchos* AF416559 APO1 *Aquila pomarina* **AF416563 SIM3 *Eusimulium securiforme*** MT276443 ANSP30286 *Zonotrichia albicollis* MT276453 ANSP30324 *Dumetella carolinensis* AY099320 ABUT/CZ/99/BUT15 *Buteo buteo* **JN006824 OA11 *Ornithomyia avicularia*** JN006826 PAS21 *Fringilla coelebs* JN006829 AGE3 *Accipiter gentilis* KT728397 NM/#9#10/AUS/2013/TA1 *Manorina melanocephala* KT728398 RHE/B30187/AUS/2015/TA1 *Xanthomyza phrygia* MH549546 KU489 *Otus sunia* MK516191 KU309 *Accipiter gularis* MK909559 KPS01F *Gallus gallus* MT276456 PRS4422 *Zonotrichia leucophrys* MT276457 PRS4423 *Passerella iliaca* MT276458 PRS4424 *Turdus migratorius* MT276460 PRS4426 *Catharus minimus* MT276462 PRS4448 *Acanthis flammea* MT276463 PRS4453 *Setophaga striata* MT276464 SCG215 *Setophaga coronata* MT276465 SCG216 *Junco hyemalis* MT276467 SCG219 *Catharus ustulatus* MT276468 SCG223 *Cardellina pusilla* MT276472 SCG227 *Melospiza lincolnii* MT276475 SCG231 *Setophaga townsendi* MT276481 SCG239 *Empidonax hammondii* MT276491 SCG257 *Loxia leucoptera* MT276496 SCG267 *Phylloscopus borealis* U39578 *Corvus frugilegus* **MT269500 *Culicoides segnis*** |
| SNS-1 | MT276438 ANSP30251 *Dumetella carolinensis* MT276446 ANSP30302 *Seiurus aurocapilla* MT276447 ANSP30303 *Hylocichla mustelina* MT276451 ANSP30315 *Poecile carolinensis* MT276454 ANSP30325 *Icterus galbula* KX179915 OZ01 *Icteria virens* MT276499 ANSP30334 *Catharus fuscescens*  MT276523 ANSP30401 *Thryothorus ludovicianus*  MT276532 ANSP30417 *Cardinalis cardinalis*  MT276479 SCG236 *Catharus ustulatus* MT276498 SCG296 *Vireo olivaceus* |
